# Network pharmacology, molecular simulation, and binding free energy calculation-based investigation of Neosetophomone B revealed key targets for the treatment of cancer

**DOI:** 10.3389/fphar.2024.1352907

**Published:** 2024-02-15

**Authors:** Abbas Khan, Yasir Waheed, Shilpa Kuttikrishnan, Kirti S. Prabhu, Tamam El-Elimat, Shahab Uddin, Feras Q. Alali, Abdelali Agouni

**Affiliations:** ^1^ Department of Pharmaceutical Sciences, College of Pharmacy, QU Health, Qatar University, Doha, Qatar; ^2^ Office of Research, Innovation, and Commercialization (ORIC), Shaheed Zulfiqar Ali Bhutto Medical University (SZABMU), Islamabad, Pakistan; ^3^ Gilbert and Rose-Marie Chagoury School of Medicine, Lebanese American University, Byblos, Lebanon; ^4^ Translational Research Institute, Academic Health System, Hamad Medical Corporation, Doha, Qatar; ^5^ Department of Medicinal Chemistry and Pharmacognosy, Faculty of Pharmacy, Jordan University of Science and Technology, Irbid, Jordan; ^6^ Dermatology Institute, Academic Health System, Hamad Medical Corporation, Doha, Qatar; ^7^ Office of Vice President for Medical and Health Sciences, Qatar University, Doha, Qatar

**Keywords:** network pharmacology, protein-protein interactions, hub gene, quantumpolarized ligand docking, molecular simulation, free energy calculation, Neosetophomone B, cancer

## Abstract

In the current study, Neosetophomone B (NSP–B) was investigated for its anti-cancerous potential using network pharmacology, quantum polarized ligand docking, molecular simulation, and binding free energy calculation. Using SwissTarget prediction, and Superpred, the molecular targets for NSP-B were predicted while cancer-associated genes were obtained from DisGeNet. Among the total predicted proteins, only 25 were reported to overlap with the disease-associated genes. A protein-protein interaction network was constructed by using Cytoscape and STRING databases. MCODE was used to detect the densely connected subnetworks which revealed three sub-clusters. Cytohubba predicted four targets, i.e., fibroblast growth factor , FGF20, FGF22, and FGF23 as hub genes. Molecular docking of NSP-B based on a quantum-polarized docking approach with FGF6, FGF20, FGF22, and FGF23 revealed stronger interactions with the key hotspot residues. Moreover, molecular simulation revealed a stable dynamic behavior, good structural packing, and residues’ flexibility of each complex. Hydrogen bonding in each complex was also observed to be above the minimum. In addition, the binding free energy was calculated using the MM/GBSA (Molecular Mechanics/Generalized Born Surface Area) and MM/PBSA (Molecular Mechanics/Poisson-Boltzmann Surface Area) approaches. The total binding free energy calculated using the MM/GBSA approach revealed values of −36.85 kcal/mol for the FGF6-NSP-B complex, −43.87 kcal/mol for the FGF20-NSP-B complex, and −37.42 kcal/mol for the FGF22-NSP-B complex, and −41.91 kcal/mol for the FGF23-NSP-B complex. The total binding free energy calculated using the MM/PBSA approach showed values of −30.05 kcal/mol for the FGF6-NSP-B complex, −39.62 kcal/mol for the FGF20-NSP-B complex, −34.89 kcal/mol for the FGF22-NSP-B complex, and −37.18 kcal/mol for the FGF23-NSP-B complex. These findings underscore the promising potential of NSP-B against FGF6, FGF20, FGF22, and FGF23, which are reported to be essential for cancer signaling. These results significantly bolster the potential of NSP-B as a promising candidate for cancer therapy.

## Introduction

Cancer is a growing major public health concern globally and has been reported to be the second leading cause of death in the United States. The characteristic features involve abnormal cellular growth with the capability of spreading to other parts of the body (Cleeland, 2000). Potential indicators and manifestations may encompass the presence of a lump, unusual bleeding, persistent cough, unexpected weight loss, and alterations in bowel movements (Woodgate et al., 2003). In 2015, approximately 90.5 million individuals worldwide were diagnosed with cancer. By 2019, the annual number of cancer cases had surged by 23.6 million, resulting in 10 million global deaths. This marked an increase of 26% and 21%, respectively, over the preceding decade. Projections for 2023 anticipate 1,958,310 new cancer cases and 609,820 deaths in the United States. Notably, prostate cancer witnessed a 3% annual rise from 2014 to 2019, countering a two-decade decline and resulting in an additional 99,000 cases (You et al., 2021; Siegel et al., 2023). The majority of cancers, 90%–95%, are attributed to genetic mutations arising from environmental and lifestyle factors, while the remaining 5%–10% are due to inherited genetics. Environmental factors encompass various non-inherited causes, including lifestyle, economic, and behavioral factors, with tobacco use (25%–30%), diet and obesity (30%–35%), infections (15%–20%), radiation (both ionizing and non-ionizing, up to 10%), lack of physical activity, and pollution being common contributors to cancer mortality. Despite its impact on cancer outcomes, psychological stress does not seem to be a risk factor for cancer onset (Berenguer et al., 2023; Yang et al., 2023).

The treatment of cancer typically involves a combination of radiation therapy, surgery, chemotherapy, and targeted therapies (Gerber, 2008). The advancement of innovative strategies in neoplastic cancer or precision drugs relies on understanding the distinct pathways and characteristics of various tumor types (Miller et al., 2019). Chemotherapy, often employed alone or alongside radiotherapy, is recognized as a highly effective treatment modality, leveraging genotoxicity to target tumor cells by generating reactive oxygen species, leading to significant tumor cell destruction (Anand et al., 2023). Hormonal treatments, widely utilized for cancer malignancies, act as cytostatic agents by impeding tumor development. This is achieved through mechanisms such as restraining hormonal growth factors, hormone receptor blockade, and limiting adrenal steroid synthesis, thus influencing the hypothalamic–pituitary–gonadal axis (HPGA) (Abraham and Staffurth, 2016).

The significance of chemotherapy in achieving cancer cures is on the rise, particularly in its application as an adjuvant to local therapies (Chu and Sartorelli, 2018). Additionally, in cases of advanced disease where the tumor has spread beyond its original site, chemotherapy plays an increasingly crucial role in alleviating cancer-related symptoms and extending life. Despite its limitations, chemotherapy remains a vital and enduring treatment approach in the field of oncology, likely retaining its importance for a substantial duration (Amjad et al., 2020). Until now many chemotherapeutic agents have been discovered for the treatment of cancer. For instance, bevacizumab in non-small cell lung cancer (NSCLC); Latrcitinib and Entrecitinib in ovarian cancer; Tazemetostate in multiple cancers; Certinib and Lorlatinib in adenocarcinoma; Trastuzumab deruxtecan in metastatic breast cancer; and Irinotecan in ovarian cancer have been discovered to target different proteins that are indispensable for the initiation and progression of cancer (Kifle et al., 2021). The emergence of gene mutations and other phenomena contribute to the resistance to the existing drugs (Khan et al., 2021; Khan et al., 2022). In the quest for effective treatments, innovative therapeutic approaches employing cutting-edge methods have proven to be valuable.

The conventional one-drug/one-target/one-disease approach to drug discovery currently faces challenges related to safety, efficacy, and sustainability. Recently, there has been a growing appreciation for network biology and polypharmacology methodologies, which involve integrating omics data and developing drugs targeting multiple pathways (Ali et al., 2022). The fusion of these approaches has given rise to a novel paradigm known as network pharmacology, which assesses the impact of drugs on both the interactome and diseasome levels. Network pharmacology utilizes computational tools to comprehensively document the molecular interactions of drug molecules within living cells. This approach proves valuable in unraveling complex relationships between botanical formulas and the entire body, enabling the identification of new drug leads, and targets, and the repurposing of existing molecules for diverse therapeutic conditions (Muhammad et al., 2018; Khan et al., 2022; Ghufran et al., 2022). Beyond expanding therapeutic options, network pharmacology analysis also strives to enhance the safety and efficacy of current medications (Sliwoski et al., 2014; Chandran et al., 2017).

Neosetophomone B (NSP-B), a meroterpenoid fungal secondary metabolite, has been recently reported to target the AKT/SKP2 axis in leukemic and multiple myeloma cell lines (Kuttikrishnan et al., 2022a; Kuttikrishnan et al., 2023a). Furthermore, NSP-B was shown to effectively inhibit FOXM1, a master regulator of the cell cycle and a transcription factor, and its downstream targets in cutaneous T-cell lymphoma and leukemia thereby paving the way for novel and safer chemotherapeutic regimens that provide a promising alternative for cancer treatment (Kuttikrishnan et al., 2022b; Kuttikrishnan et al., 2023b). Considering the anti-cancerous potential of NSP-B, the current study uses network pharmacology combined with quantum-polarized ligand docking (QPLD) and molecular simulation to discover novel targets for NSP-B. Furthermore, binding free energy was calculated for the top hub genes-NSP-B complexes. This study will guide the selective inhibition of cancer targets in the clinical trials.

## Materials and methods

### Targets prediction for NSP-B

**FIGURE 1:**
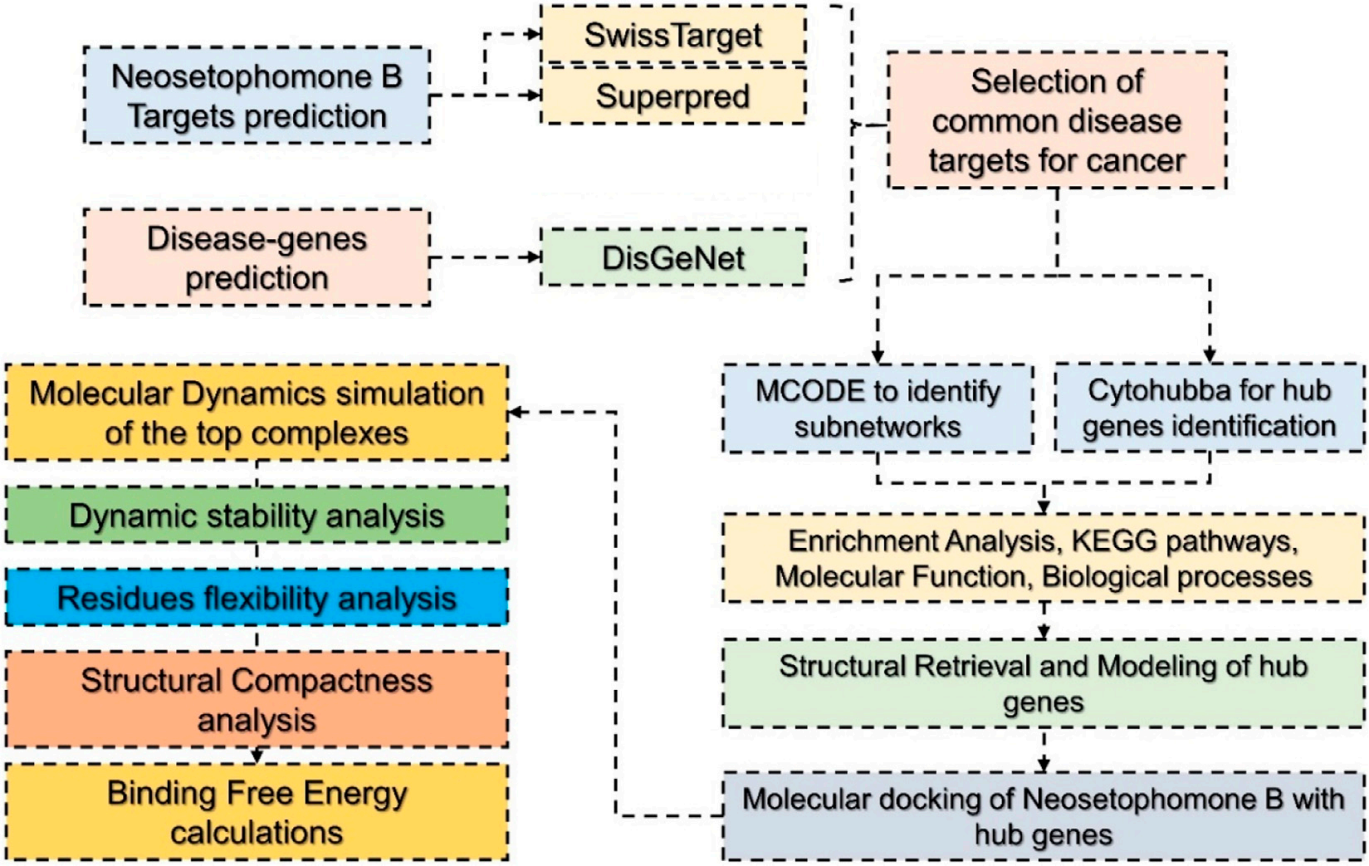
Hierarchical workflow of the study involving various steps from target prediction to target retrieval, PPI construction, identification of hub genes, molecular docking, and molecular simulation.

To predict targets for NSP-B (Compound CID: 146683131), we used three different databases. SMILES of NSP-B were submitted as the input and targets were predicted using SwissTarget Prediction (http://www.swisstargetprediction.ch/) (Daina et al., 2019), and Superpred (https://prediction.charite.de/) (Gallo et al., 2022). The disease-related genes were obtained from DisGeNet (https://www.disgenet.org/search) by searching the term “cancer” to retrieve all the disease-related proteins/genes associated with cancer (Piñero et al., 2016). Among the predicted targets and the disease-associated targets, the common targets were selected for the PPI network construction. The methodological workflow is summarized in Figure 1.

### Construction of PPI network

The construction of the PPI network for NSP-B’s candidate targets against cancer was achieved using the STRING database (https://string-db.org/cgi/input?sessionId=btWeOUvPdvTt&input_page_active_form=single_identifier) with parameters set at the highest confidence level (0.900) (Szklarczyk et al., 2021; Doncheva et al., 2022). Subsequently, the resulting PPI network was imported into Cytoscape v3.8.2 for subnetwork identification and core target screening, employing the MCODE plugin with specific parameters: “Degree Cutoff = 2, Node Score Cutoff = 0.2, and K-Core = 2”. The top 4 core targets were then selected based on the Cytohubba analysis (Lotia et al., 2013; Chin et al., 2014; Otasek et al., 2019).

### Structural retrieval and quantum-polarized ligand docking (QLPD)

The available crystallographic coordinates were retrieved from RCSB while the non-available coordinates were modeled using Alpha Fold 2.0 (Burley et al., 2019; Jumper et al., 2021). Each structure was prepared using the protein preparation wizard in the Schrodinger Maestro (Maestro et al., 2020). The structures were pre-processed by using the default setting while refined by using the pH 7.0 and OPLS 2.1 force field for minimization. Restrained minimization was carried out where the convergence of heavy atoms to RMSD was set to 0.30 Å. The ligand molecule was downloaded from PubChem and minimized by using the MMFFx force field. For the binding site detection sitemap module was used. Advanced docking methods, including scoring functions, aim to estimate binding energies, providing quantitative insights into ligand-protein interactions (Ferreira et al., 2015). To determine the activity of NSP-B against the selected targets we also used the quantum-polarized ligand docking (QPLD) approach which is the most accurate method in evaluating the binding potential of small molecules by combining the quantum mechanical and molecular mechanics properties (Cho et al., 2005). This approach provides a more accurate description of the electronic interactions between the ligand and the protein, taking into account the polarization effects that occur due to the charge distribution in the ligand and the protein than the traditional docking methods. It uses the density functional theory (DFT) or semi-empirical methods properties to quantify the protein and ligand properties. We used QPLD approaches by considering the ligand vdW scaling as 0.8, RMSD deviation less than 0.5 while a maximum of 10 poses were allowed using the Schrodinger Maestro software. We used Jaguar for the QM charges assignment while the re-docking was performed by employing the XP approaches with the maximum atomic displacement of 1.3 Å. The best pose was then visualized in PyMOL for molecular interactions analysis (DeLano, 2002).

### All-atoms simulation in explicit solvent

To perform molecular simulations of all the systems the coordinates, and topology files were prepared using the “tLeap” an integrated module in AMBER21 (Case et al., 2005; Salomon-Ferrer et al., 2013). A solvent box (OPC) optimal point charge was added around each system, and ions were added to neutralize the charge. The ligand molecule was parameterized by using the GAFF2 force file while the initial topology and frcmod file was generated with antechamber and parmchk2. Next, each system underwent energy minimization using a minimization algorithm such as steepest descent and conjugate gradient. The minimization process continued until the system reached a convergence criterion, such as a maximum force or energy change threshold. To allow each system to reach the desired simulation temperature and equilibrate, a temperature coupling algorithm (such as Langevin Dynamics or Berendsen thermostat) was used to gradually heat the system from a low temperature. Long-range electrostatic interactions were calculated using the Particle Mesh Ewald (PME) method, while van der Waals forces were calculated using Lennard-Jone’s potential (Toukmaji et al., 2000). Each system was equilibrated at the target temperature and pressure for a certain period of time in several stages, including positional restraint, slow heating, and equilibration without restraints. To maintain covalent bond lengths, the SHAKE algorithm was used to constrain bond lengths and angles. The pressure of the system was controlled using a barostat such as Berendsen or Andersen (Fyta, 2016). After equilibration, each system was simulated for a production time of 300 ns using a molecular dynamics algorithm such as NPT or NVT ensemble (Salomon-Ferrer et al., 2013). In this step, simulation parameters including time step and cut-off distances were set. Finally, the trajectory obtained from the production simulation was analyzed using CPPTRAJ or PTRAJ modules (Roe and Cheatham, 2013). We calculated RMSD, RMSF, Rg, and hydrogen bonding for each system (Cooper, 1976; Maiorov and Crippen, 1994; Lobanov et al., 2008).

$$RMSD=\sqrt{\frac{\sum d^{2}i=1}{N_{atoms}}}$$ (ii)

Where:

d_i_ is the difference of position between atoms and *i* refers to the original and superimposed structure. Whereas the root mean square fluctuation (RMSF) can be computed by employing B-factor (Chin et al., 2014), which is the most imperative constraint to compute the flexibility of all the residues in a protein. Mathematically the RMSF can be calculated by using the following equation.

$$Thermal\;factor\;or\;B-factor=\left[\left(8\pi **2\right)/3\right]\;\left(msf\right)$$ (iii)

The radius of gyration measures the compactness of a protein structure.

$$R_{gyr}^{2}=\frac{1}{M}\;\sum_{i=1}^{N}m_{i}\left(r_{i}-R^{2}\right)$$ (iv)

where;

$$M=\sum_{i=1}^{N}m_{i}$$ (v)

is the total mass and;

$$R=N^{-1}\sum_{i=1}^{N}r_{i}$$ (vi)

is the center of mass of the protein consisting of N atoms.

### Binding free energy estimation through MM/GBSA and MM/PBSA analysis

Insights into the process of how a protein identifies its biologically significant ligand or a small molecule inhibitor significantly impact the discovery of effective small molecule treatments. This approach has the advantage over others as it is less time-consuming and computationally inexpensive (Chen et al., 2016). It has been widely used to determine the BFE for protein-protein and protein-ligand complexes. We calculated the BFE for each complex (*G*_*complex*_ *,*_*solvated*_) and the unbound states of NSP-B (*G*_*NSP-B, solvated*_) and receptors (*G*_*receptors, solvated*_). AMBER utilizes the MM/GBSA (Molecular Mechanics/Generalized Born Surface Area) methodology for binding free energy calculations. This approach integrates molecular mechanics force fields, a generalized Born (GB) implicit solvent model, and a surface area term. The MMPBSA.py module within AMBER conducts the computation, with essential parameters encompassing the molecular dynamics trajectory, force field parameters, and implicit solvent specifications. The MM/GBSA technique in AMBER presents a reliable computational framework for the estimation of binding free energies in biomolecular systems (Chen et al., 2016). The following equation was used to calculate each term in the total binding energy.

$${∆G}_{bind}=G_{\left(complex,solvated\right)}-\;G_{\left(Neosetophomone\;B,solvated\right)}-\;G_{\left(receptors,solvated\right)}$$ (vii)

This equation can be used to determine the contribution of interaction in the complex and can be expressed as;

$$G=E_{Molecular\;Mechanics}-\;G_{solvated}-\;TS$$ (viii)

This equation can be further restructured to calculate the specific energy term.

$${∆G}_{bind}={∆E}_{Molecular\;Mechanics}+{∆G}_{solvated}-∆TS={∆G}_{vaccum}+{∆G}_{solvated}$$ (ix)

$${∆E}_{Molecular\;Mechanics}={∆E}_{\mathrm{int}}+{∆E}_{electrostatic}+{∆E}_{vdW}$$ (x)

$$∆G_{solvated}=∆G_{Generalized\;born}+∆G_{surface\;area}$$ (xi)

$$∆G_{surface\;area}=\gamma .SASA+b$$ (xii)

$$∆G_{vaccum}={∆E}_{Molecular\;Mechanics}-T∆S$$ (xiii)

The total binding energy is a composite of various components. Specifically, the free energy linked to the binding of ligand-protein, PPI, or protein-nucleic acid is referred to as ΔG_bind_. The cumulative gas phase energy, including ΔE_internal_, ΔE_electrostatic_, and ΔE_vdw_, is denoted as ΔEMM. Solvation effects contribute through the combination of polar (ΔG_PB/GB_) and nonpolar (ΔG_SA_) components. Here, ΔG_PB/GB_ represents the polar contribution calculated using Poisson–Boltzmann (PB) or generalized Born (GB) methods, while ΔGSA is the nonpolar solvation free energy, often determined through a linear function of solvent-accessible surface area (SASA). The conformational binding entropy, typically evaluated through normal-mode analysis, is expressed as -TΔS. However, the computation of conformational entropy was omitted due to computational expense and associated inaccuracies. In MM/PBSA and MM/GBSA, ΔE_internal_ consistently remains zero in single trajectory complex calculations (Nadeem et al., 2023).

## Results and discussion

### Drug and disease-related target retrieval

**FIGURE 2:**
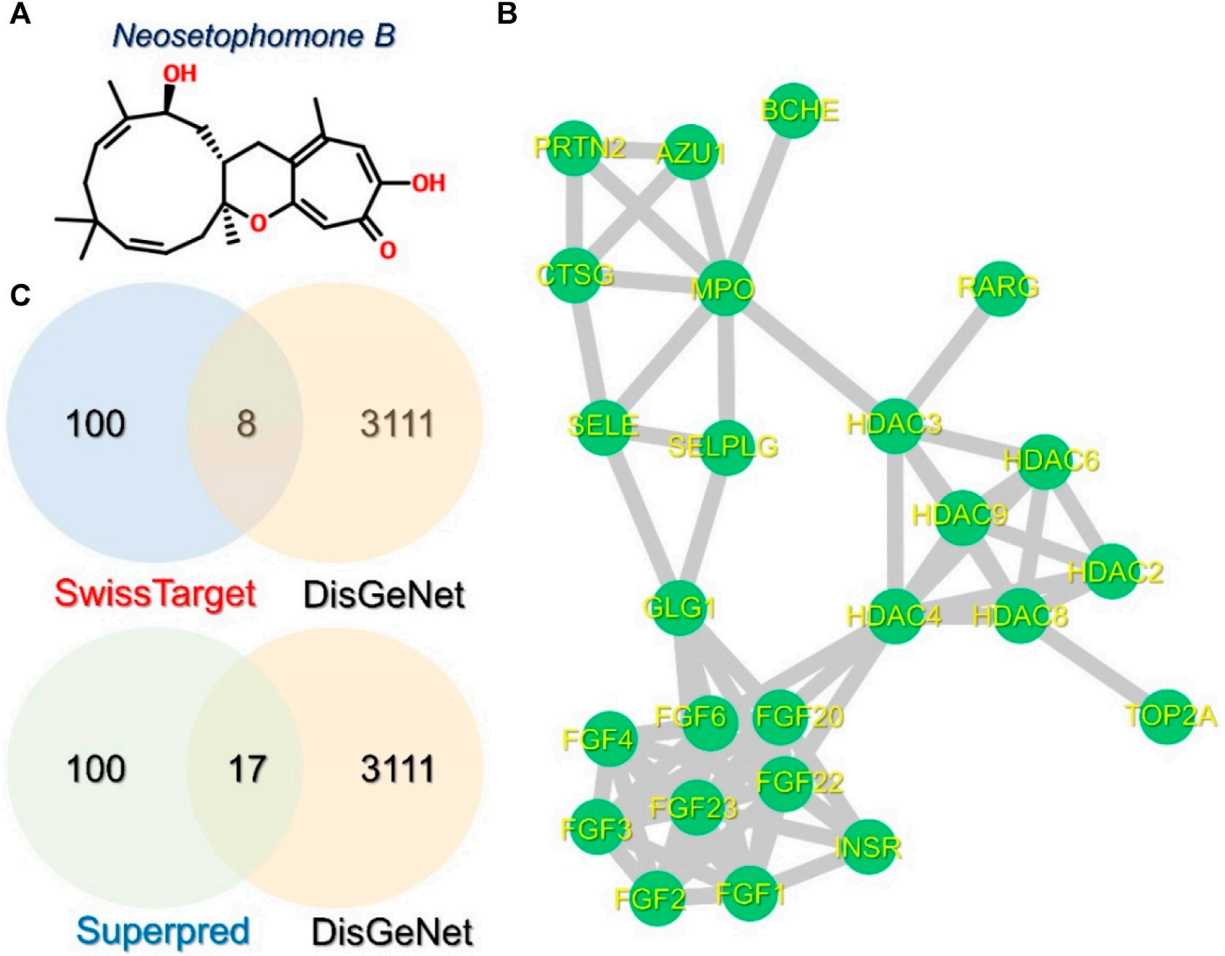
Structure of NSP-B, Venn diagrams, and PPI of the selected compound and proteins are shown. **(A)** shows the 2D structure of NSP-B, **(B)** shows the PPI network of the 25 common proteins in the selected databases, and **(C)** shows the common genes identified between the predicted and disease-associated targets in breast cancer.

In order to investigate the mechanism of interaction of NSP-B with the key cancer targets, different databases were used for retrieval of drug and disease-related targets. The structure of NSP-B was obtained from PubChem and targets were retrieved from various databases. A total of 100 targets were retrieved for this drug in the SwissTarget database while Superpred returned 76 targets. With regards to disease-associated genes, a total of 3,111 disease genes were predicted as cancer biomarkers in DisGeNet database. Among these, 8 and 17 genes were common with targets retrieved in the SwissTarget and Superpred databases, respectively. A PPI network of these 25 common proteins was then constructed using the STING protein database and imported into Cytoscape. The 2D structure of NSP-B is shown in Figure 2A, while the Venn diagrams for the predicted targets and disease-associated targets are provided in Figure 2B. The PPI network of the common 25 targets was constructed and is depicted in Figure 2C.

### Identification of small subnetworks and hub genes

**FIGURE 3:**
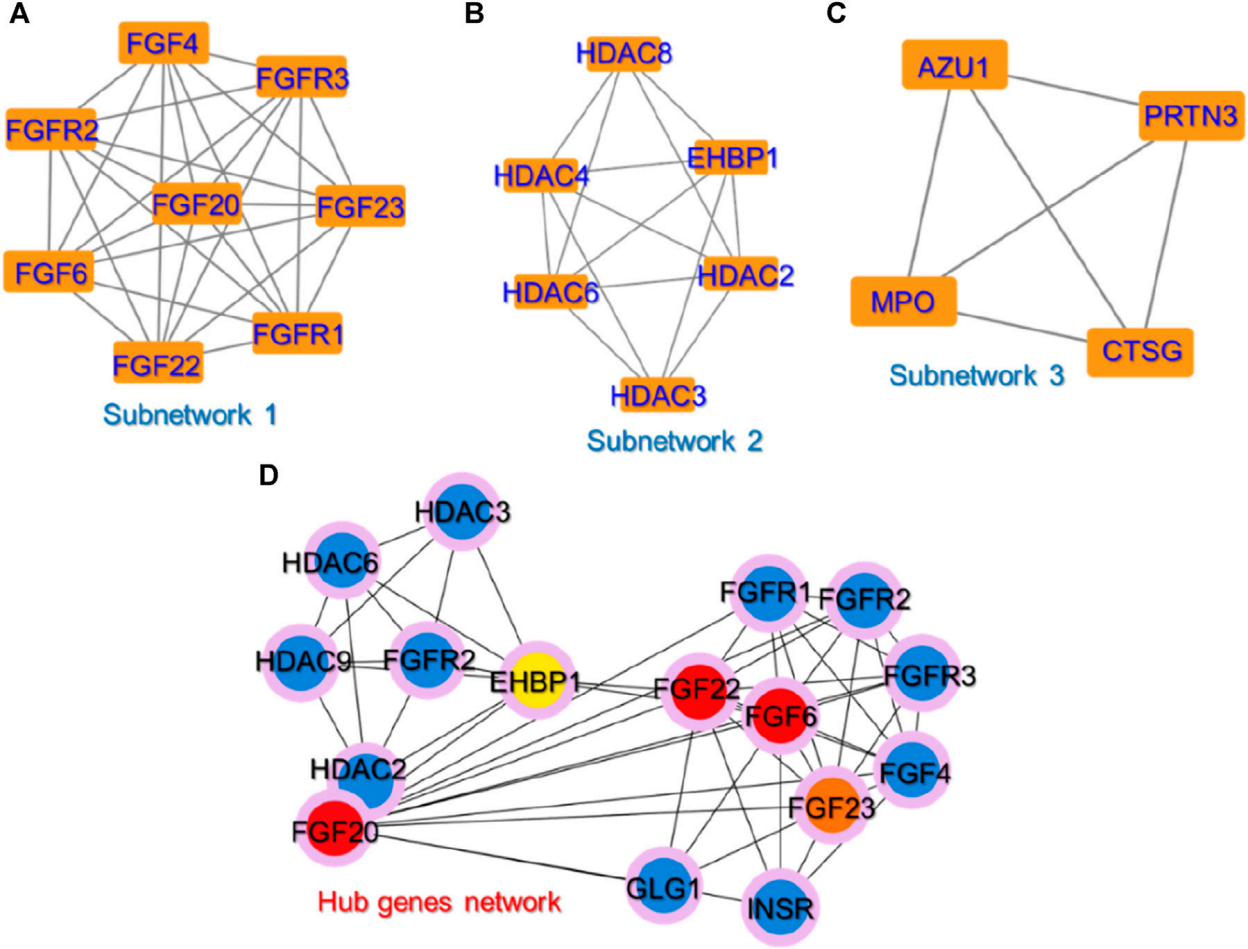
The identified sub-clusters and hub genes networks from the whole PPI network. **(A–C)** shows the top three sub-networks in the whole PPI network, while **(D)** shows the key hub genes in the PPI network depicted in red, orange, and yellow colors. The blue-colored genes represent the sub-nodes that interact with these hub genes.

Identifying small subnetworks in PPI networks using tools like MCODE (Molecular Complex Detection) in Cytoscape offers valuable insights into the organization and functionality of biological systems. These subnetworks represent functional modules or clusters of deeply associated proteins that are essential for certain cellular processes such as signaling cascades, metabolic pathways, or protein complexes. Understanding the organization of proteins into functional modules provides insights into the underlying biological processes. Furthermore, subnetworks usually exhibit proteins that are associated with specific diseases or pathological conditions and thus the identification of such subnetworks can contribute to the understanding of disease mechanisms and act as therapeutic biomarkers for a particular disease. Hence, we also used the MCODE module to identify the subnetworks in the PPI network. Three small subnetworks were identified. In the first subnetwork, *FGFR1* (Fibroblast growth factor receptor 1), *FGFR2*, *FGFR3*, *FGF4*, *FGF6*, *FGF20*, *FGF22*, and *FGF23* were clustered. In the second subnetwork, *EHBP1* (EH domain-binding protein 1), *HDAC2* (Histone deacetylase 2), *HDAC3*, *HDAC4*, *HDAC6*, and *HDAC8* were clustered while in the third subnetwork, *AZU1* (Azurocidin 1), *PRTN3* (Proteinase 3), *MPO* (Myeloperoxidase), and *CTSG* (Cathepsin G) were clustered. The subnetworks are shown in Figures 3A–C.

To predict the hub genes in the PPI network of 25 proteins, Cytohubba was used. Among the 25 common proteins, only five proteins were identified as hub genes based on the degree and are presented in Figure 3D. Among the hub genes identified FGF6, FGF20, FGF22, FGF23, and EHBP1 were identified as the key biomarker genes. The FGF signaling network is ubiquitous in normal cell growth, survival, differentiation, and angiogenesis, but it has also been associated with cancer development. FGFs’ capacity to promote tumor growth is highly dependent on specific FGFR signaling. FGF can overcome chemotherapy resistance by boosting tumor cell survival, implying that chemotherapy may be more effective when combined with FGF inhibitor treatment. Previous studies have demonstrated that FGFs stimulate the growth and invasion of numerous cancer types including non-small lung cells, hepatocellular carcinoma (HCC), melanomas, astrocytoma, breast, pancreatic, bladder, head and neck, and prostate cancers making the FGF signaling pathway a promising target for cancer therapy (Korc and Friesel, 2009; Ao et al., 2015; Ropiquet et al., 2000; Francavilla and OBrien, 2022; Katoh, 2016). FGFs are reported to be essential for cancer signaling (Ferguson et al., 2021). For instance, the increased expression of FGF6 has been reported by previous studies in different types of cancers particularly breast cancer (Ropiquet et al., 2000; Francavilla and OBrien, 2022). Another study reported that targeting the FGFR proteins using the inhibitors acts as a starting point for the promising cancer therapy (Katoh, 2016). Moreover, EHBP1 has been reported to be a well-validated target in prostate cancer (Kolawole, 2012; Ao et al., 2015). This further supports the validity of these selected hub genes as potential targets for the treatment of cancer.

### Quantum-polarized ligand docking of NSP-B with the hub genes

**FIGURE 4:**
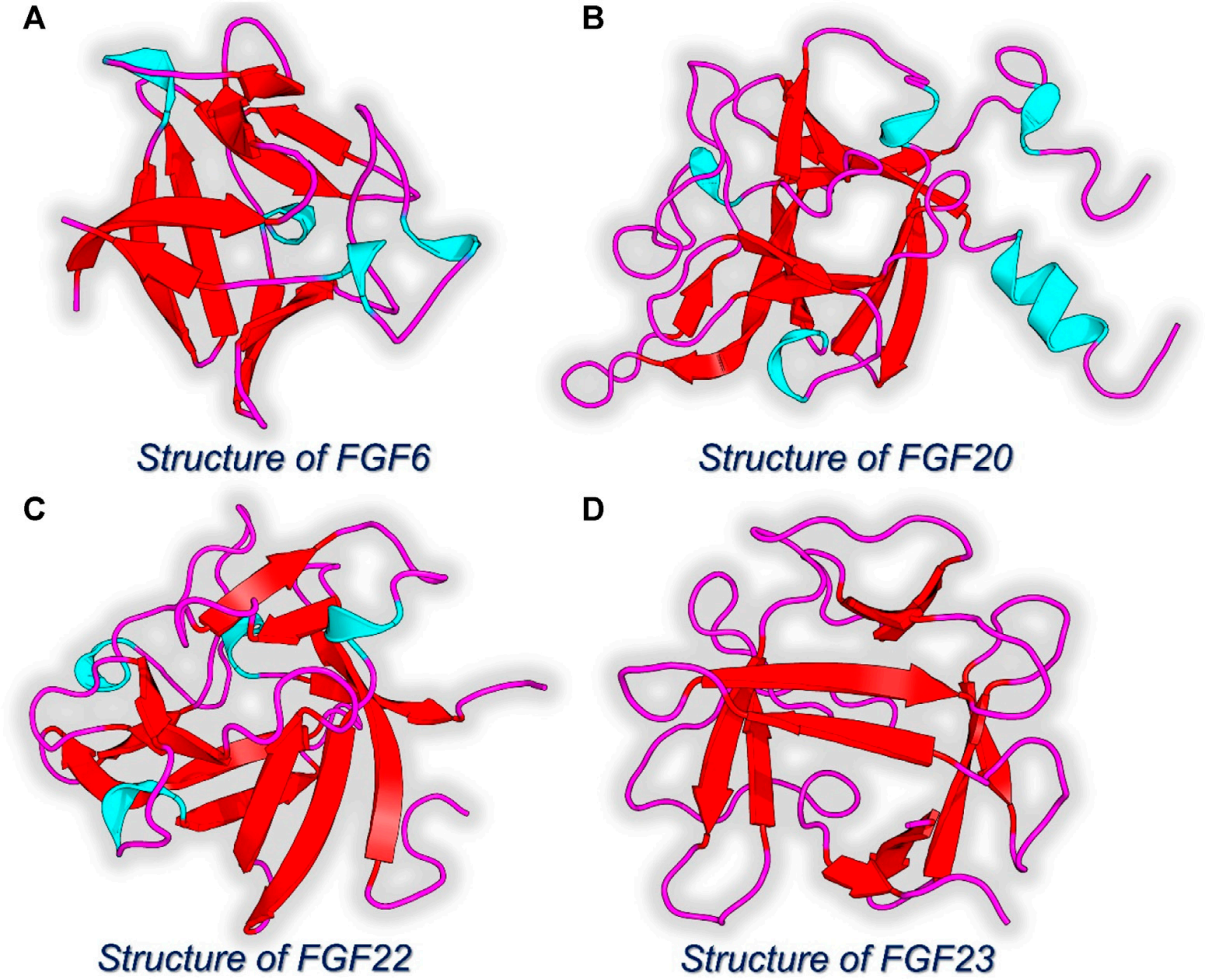
3D structures of the hub genes identified as the key targets for NSP-B. **(A)** Structure of FGF6. **(B)** Structure of FGF20. **(C)** Structure of FDF22. **(D)** Structure of FGF23.

Since the role of FGF family proteins is obvious in various cancers, the top four FGF proteins acting as hub genes were selected for the interaction with NSP-B using the 3D structures of the target proteins retrieved from Protein databank, and active sites were identified using the sitemap tool in Schrodinger Maestro. The 3D structures of each selected protein, i.e., FGF6, FGF20, FGF22, and FGF23 are given in Figures 4A–D.

**FIGURE 5:**
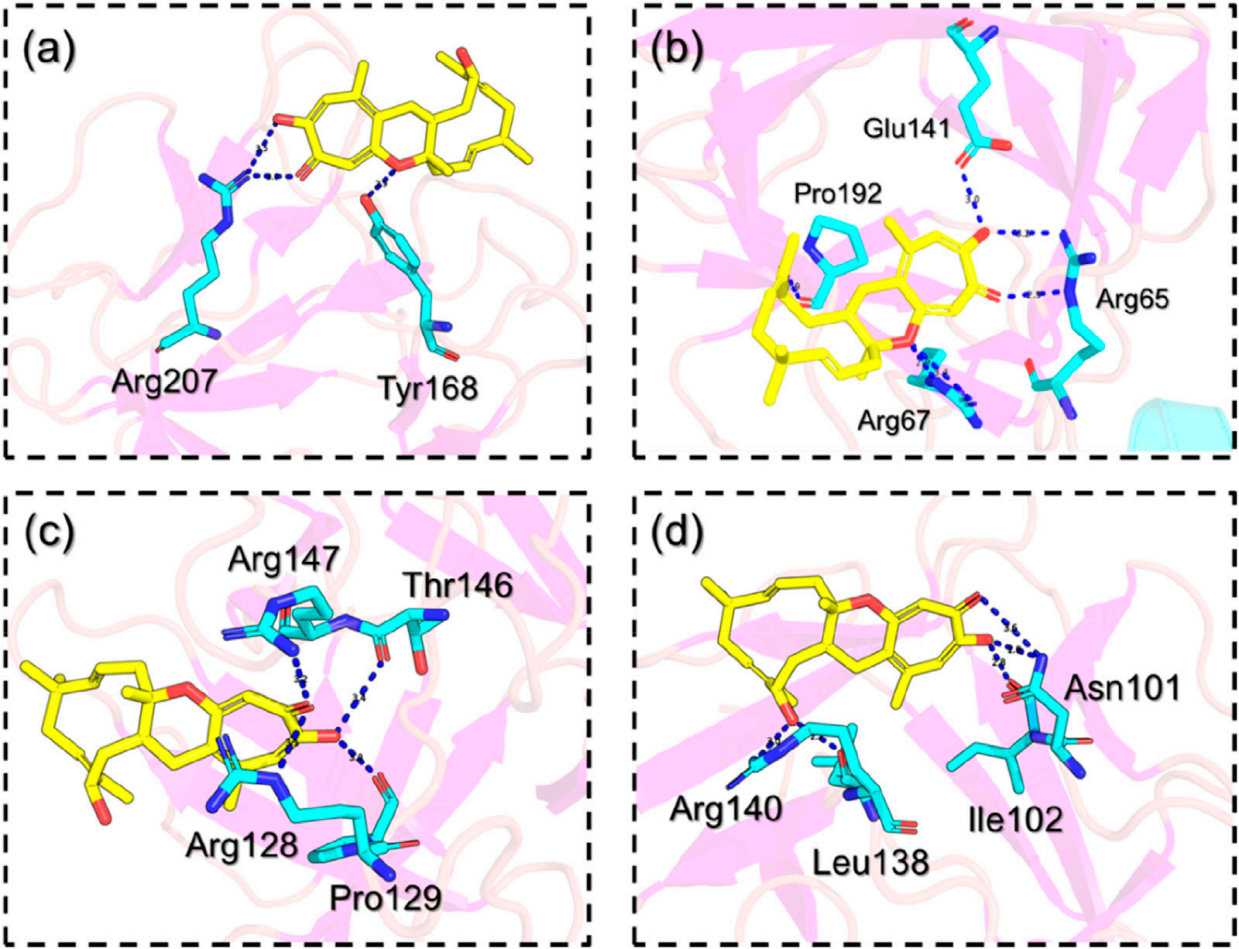
Interaction pattern of the selected hub genes with NSP-B. **(A)** shows the interaction pattern of NSP-B with FGF6, **(B)** shows the interaction pattern of NSP-B with FGF20, **(C)** shows the interaction pattern of NSP-B with FGF22 while **(D)** shows the interaction pattern of NSP-B with FGF23.

Using the QPLD approach, FGF6 in complex with NSP-B reported a docking score of −7.89 kcal/mol with three hydrogen bonds in the complex. Among the hydrogen bonds, Arg207 established two hydrogen bonds while Tyr168 reported a single hydrogen bond. This shows the binding potential of NSP-B towards FGF6. The interaction pattern of NSP-B-FGF6 is shown in Figure 5A. On the other hand, FGF20 in complex with NSP-B reported a docking score of −10.75 kcal/mol with several hydrogen bonds with the key residues. The interactions involve Arg65 with two hydrogen bonds, Arg67 established a single hydrogen bond, and Glu141 and Pro192 also reported single hydrogen bonds. The binding pattern for the FGF20-NSP-B complex is given in Figure 5B. The FGF22-NSP-B complex reported a docking score of −9.61 kcal/mol with the four hydrogen bonds in the interaction paradigm. As given in Figure 5C, amino acids such as Arg128, Pro129, Thr146, and Arg147 are involved in creating the hydrogen bonds. This also shows the binding potential of this molecule towards diverse proteins. Unlike the others, the FGF23- NSP-B complex reported five hydrogen bonds with the highest docking score of −11.24 kcal/mol. The hydrogen bonding involves Asn101, ile102, leu138 and Arg140. The interaction pattern for the FGF23-NSP-B complex is given in Figure 5D. This consistent interaction pattern with different proteins highlights the potential for the ligand to selectively target and modulate the activity of this class of proteins. The observed multi-protein hydrogen bonding reinforces the ligand’s potential as a versatile and promising candidate for therapeutic development against a range of closely related targets.

### Dynamic stability analysis of the complexes

**FIGURE 6:**
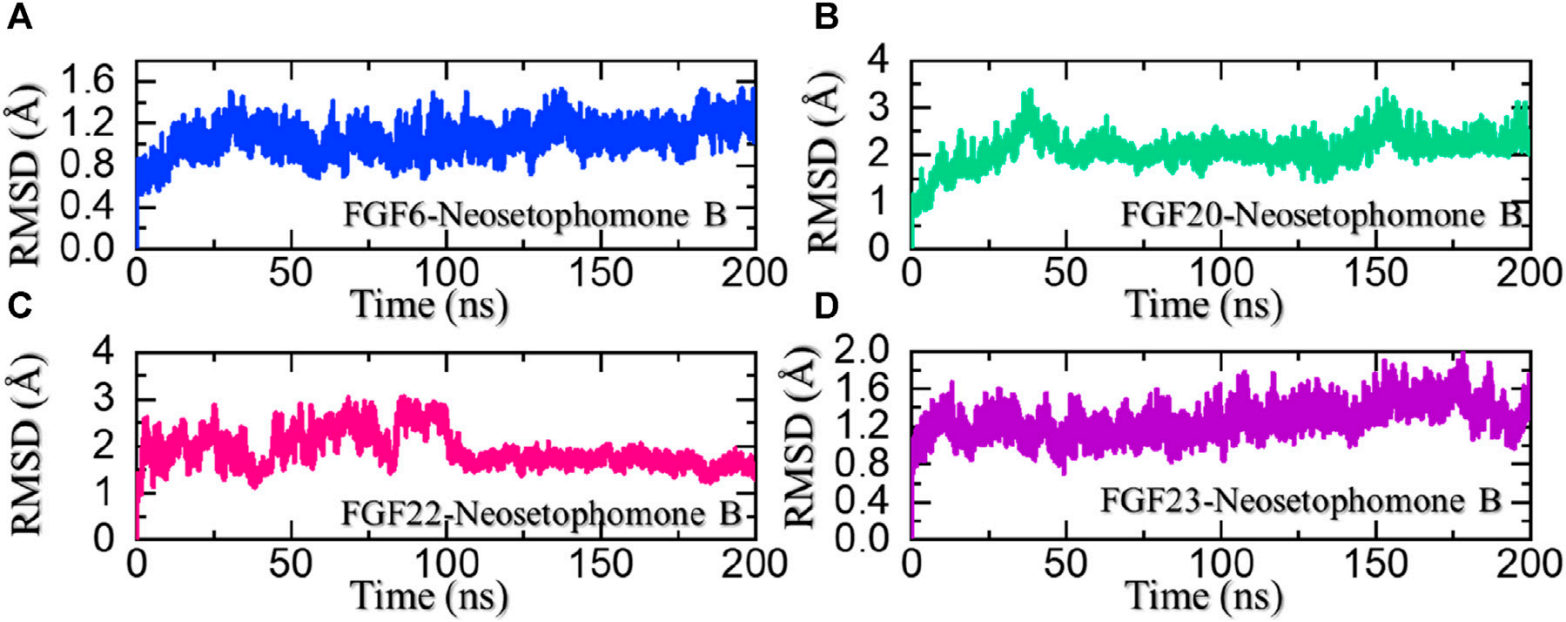
Dynamic stability analysis of the NSP-B bound complex with the selected hub genes. **(A)** shows the RMSD for the NSP-B-FGF6 complex, **(B)** shows the RMSD for the NSP-B-FGF20 complex, **(C)** shows the RMSD for the NSP-B-FGF22 complex while **(D)** shows the RMSD for the NSP-B-FGF23 complex during the simulation.

Dynamic stability investigation determines the pharmacological potential of the ligand-bound complex during the simulation. It is an essential parameter in deciphering essential knowledge regarding the binding stability of the drug to its target. To determine the stability variation of these complexes we also calculated root mean square deviation (RMSD) as a function of time. It can be seen that the FGF6-NSP-B stabilized at 1.0 Å and maintained a similar level throughout the simulation. The complex reported no significant structural perturbation and therefore demonstrated the stable binding of NSP-B with FGF6 during the simulation. The RMSD results for the NSP-B-FGF6 are given in Figure 6A.

On the other hand, the FGF20-NSP-B complex reported two minor deviations at 40 and 150 ns. With no significant structural perturbation, the complex stabilized at 2.0 Å and thereafter demonstrated a stable dynamic behavior. The RMSD results for the NSP-B-FGF20 are given in Figure 6B. In the case of FGF22-NSP-B, the complex initially reported significant dynamic instability but after 100 ns the RMSD of the complex decreased and stabilized. The structure attained stability after 100 ns and maintained a uniform RMSD pattern until the end of the simulation. The RMSD results for the NSP-B-FGF22 are given in Figure 6C. Moreover, the FGF23-NSP-B complex reported a dynamically stable behavior with no significant structural perturbation indicating the binding stability of NSP-B with FGF23. The RMSD results for the NSP-B-FGF23 complex are given in Figure 6C. These ligand-bound complexes exhibiting stable RMSD with minimal perturbation throughout simulation time suggest a robust and energetically favorable binding interaction. This steadfast structural stability implies that the ligand maintains a consistent and well-defined conformation within the binding site, reinforcing the reliability of the ligand-protein complexes. Furthermore, this unyielding stability indicates a promising foundation for the development of a pharmacologically effective molecule, with the potential for sustained and reliable interactions, enhancing its candidacy for further drug development endeavors.

### Structural compactness analysis

**FIGURE 7:**
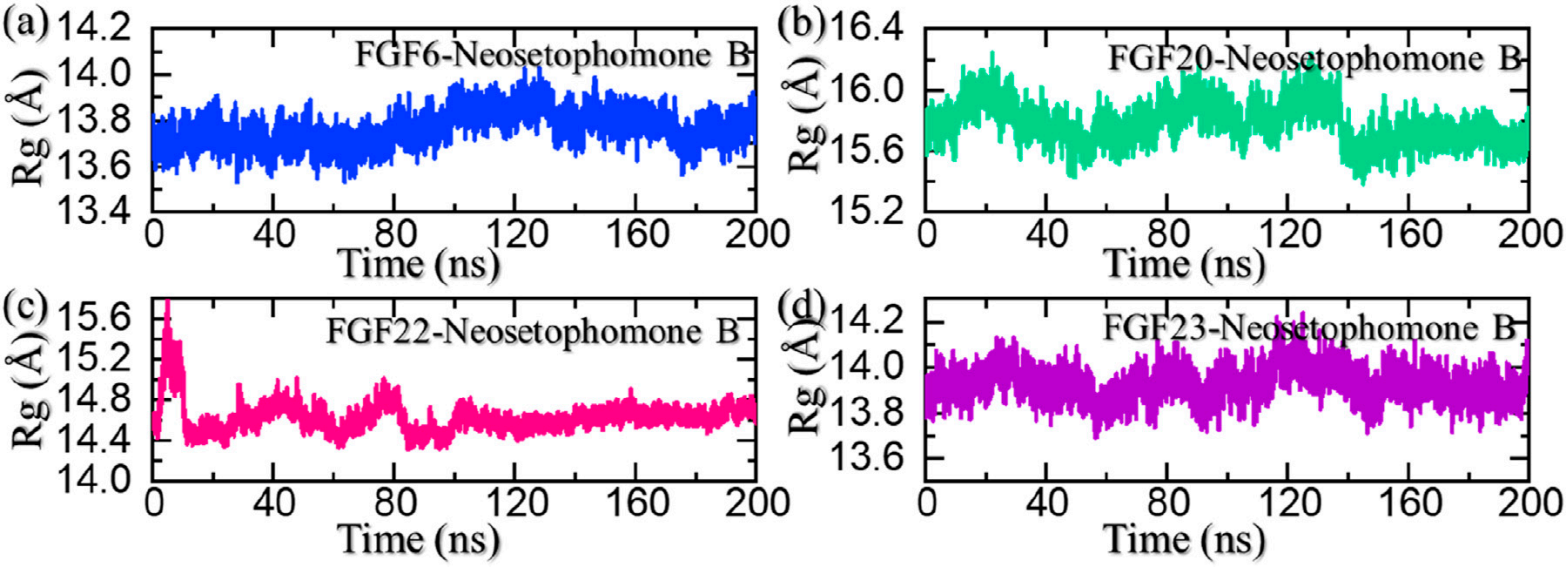
Structural compactness analysis of the NSP-B bound complex with the selected hub genes. **(A)** shows the Rg for the NSP-B-FGF6 complex, **(B)** shows the Rg for the NSP-B-FGF20 complex, **(C)** shows the Rg for the NSP-B-FGF22 complex while, **(D)** shows the Rg for the NSP-B-FGF23 complex during the simulation.

The radius of gyration (Rg) serves as a measure of the compactness or structural stability of a ligand-protein complex during molecular dynamic simulations. A consistent or decreasing Rg over the simulation duration indicates that the complex maintains a compact and well-defined conformation. In the context of ligand pharmacological potential, a stable or decreasing Rg suggests that the ligand forms a persistent and compact binding interface, reinforcing its structural integrity and potential for pharmacological efficacy by maintaining a stable interaction with the target protein. We also calculated Rg as a function of time using the simulation trajectories. As shown in Figure 7A, the FGF6-NSP-B complex maintained a stable compact topology throughout the simulation. The size of the receptor increased a little between 80 and 160 ns; however, then decreased back and maintained a level at 13.70 Å. This shows the compact nature and stabilized binding of the protein-ligand complex during the simulation. On the other hand, the Rg for the FGF20-NSP-B started from 15.75 Å and demonstrated a wave-like pattern where an increase and decrease in the Rg levels were observed until 125 ns. Afterward, the Rg level decreased abruptly and maintained a lower level at 15.50 Å. The Rg pattern for the FGF20-NSP-B is given in Figure 7B. In the case of the FGF22-NSP-B complex, the Rg level abruptly increased initially and then decreased back at 15 ns. Afterward, the Rg level was maintained at the same level with no notable variation in values. The Rg pattern for the FGF22-NSP-B is given in Figure 7C.

On the other hand, the FGF23-NSP-B complex maintained an Rg level of 14.0 Å with no significant variation thus showing a uniform protein size during the simulation. The Rg pattern for the FGF23-NSP-B is given in Figure 7D. In sum, the Rg results show that these protein-ligand complexes maintained a compact topology with minimal unbinding events throughout the simulation and thus show the pharmacological potential of this molecule against these targets.

### Residue’s flexibility analysis

**FIGURE 8:**
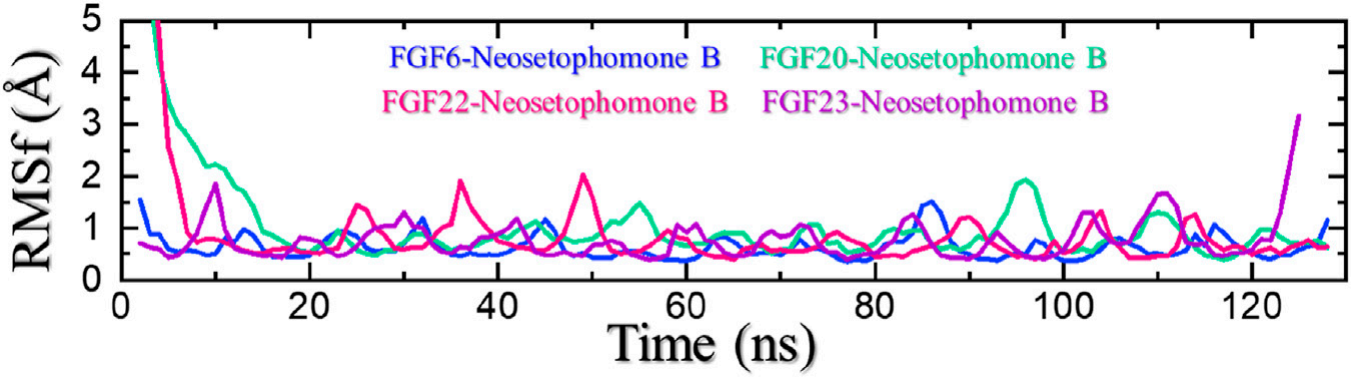
Residues’ flexibility analysis of the NSP-B bound complexes.

In molecular dynamic (MD) simulations, the root mean square fluctuation (RMSF) is a useful metric and can be used to compare the flexibility of different regions within a molecule or between different molecules. This can help identify flexible regions that may be important for ligand binding or PPI interactions. RMSF is also an important parameter for validating MD simulations. Experimental measurements of RMSF can be used to validate the accuracy of the simulation and the force field used. A good agreement between the experimental and simulated RMSF values indicates that the simulation is accurately capturing the flexibility and dynamics of the biomolecule. All the complexes demonstrated minimal fluctuations except for FGF22-NSP-B which demonstrated the highest fluctuations. This shows that the internal fluctuation is stabilized by the binding of NSP-B and therefore produces the potential pharmacological properties. The RMSF for each complex is given in Figure 8.

### Hydrogen bonding analysis

**FIGURE 9:**
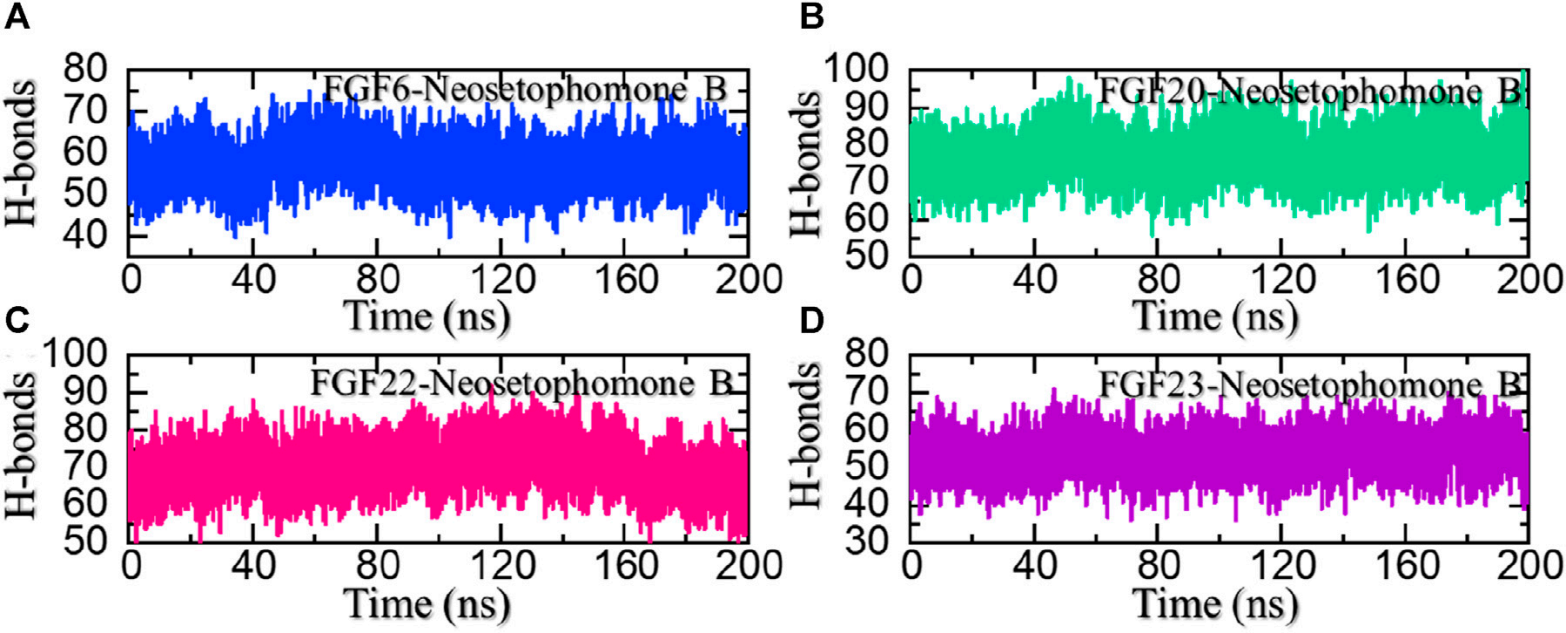
Hydrogen bonding (H-bonds) analysis of the NSP-B bound complex with the selected hub genes. **(A)** shows the H-bonds for the NSP-B-FGF6 complex, **(B)** shows the H-bonds for the NSP-B-FGF20 complex, **(C)** shows the H-bonds for the NSP-B-FGF22 complex while **(D)** show the H-bonds for the NSP-B-FGF23 complex during the simulation.

Hydrogen bonds, especially in the realm of protein-ligand interactions, play a pivotal role in gauging the strength of binding interactions. They constitute a crucial element in unraveling the intricacies of diverse biological processes, understanding disease mechanisms, and assessing how mutations influence protein coupling and molecular signaling. Given the fundamental significance of hydrogen bonding in these processes, we quantified the number of hydrogen bonds in each trajectory across different time points, providing insights into the dynamic nature of these vital interactions. Considering the importance of hydrogen bonding calculation in the binding strength of the protein-ligand complex, we also calculated the average number of hydrogen bonds in each complex. In the FGF6-NSP-B complex, the average number of hydrogen bonds was calculated to be 62. In the FGF20-NSP-B complex, the average number of hydrogen bonds was calculated to be 82. In the FGF22-NSP-B complex, the average number of hydrogen bonds was 72, while in the FGF23-NSP-B complex, the average number of hydrogen bonds was 55 in number. The hydrogen bond graphs are shown in Figures 9A–D.

### Binding free energy calculation

Validation of the docking results can be performed by using the binding free energy calculation approach which is an accurate, fast, and computationally inexpensive approach. This approach has been widely employed to determine the binding potential of various protein complexes in different diseases. Therefore, considering the potential of this approach, we also calculated the binding free energy using the MM/GBSA and MM/PBSA methods. Using the MM/GBSA and MM/PBSA methods, the vdW was calculated to be −33.84 kcal/mol for the FGF6-NSP-B complex, −39.07 kcal/mol for the FGF20-NSP-B complex, −36.87 kcal/mol for the FGF22-NSP-B complex, and −40.25 kcal/mol for the FGF23-NSP-B complex. On the other hand, the electrostatic energy was calculated to be −5.76 kcal/mol for the FGF6-NSP-B complex, −6.41 kcal/mol for the FGF20-NSP-B complex, −3.87 kcal/mol for the FGF22-NSP-B complex, and −4.17 kcal/mol for the FGF23-NSP-B complex. Using the MM/GBSA approach, the total free binding energy was calculated to be −36.85 kcal/mol for the FGF6-NSP-B complex, −43.87 kcal/mol for the FGF20-NSP-B complex, −37.42 kcal/mol for the FGF22-NSP-B complex, −41.91 kcal/mol for the FGF23-NSP-B complex. The binding free energy results using the MM/GBSA approach are given in Table 1.

**TABLE 1:** Binding free energy calculation results using the MM/GBSA approach. The results are provided in kcal/mol.

MM/GBSA				
Parameters	FGF6	FGF20	FGF22	FGF23
vdW	−33.84	−39.07	−36.87	−40.25
Electrostatic Energy	−5.76	−6.41	−3.87	−4.17
EGB	4.21	5.24	6.21	4.28
ESURF	−1.46	−3.63	−2.89	−1.77
Total Binding Energy	−36.85	−43.87	−37.42	−41.91

The MM/PBSA approach was also used to estimate the binding free energy and revealed similar results for vdW and electrostatic energies as those found with MM/GBSA approach while variations in the total binding free energy were observed. The total binding free energy using the MM/PBSA approach revealed values of −30.05 kcal/mol for the FGF6-NSP-B complex, −39.62 kcal/mol for the FGF20-NSP-B complex, −34.89 kcal/mol for the FGF22-NSP-B complex, while the FGF23-NSP-B complex demonstrated a value of −37.18 kcal/mol. Overall, these results demonstrate that NSP-B exhibits excellent pharmacological properties against FGF6, FGF20, FGF22, and FGF23. This further supports the potential of NSP-B as a promising anti-cancer therapy. The binding free energy results using the MM/PBSA approach are summarized in Table 2.

**TABLE 2:** Binding free energy calculation results using the MM/PBSA approach. The results are provided in kcal/mol.

MM/PBSA				
Parameters	FGF6	FGF20	FGF22	FGF23
vdW	−33.84	−39.07	−36.87	−40.25
Electrostatic Energy	−5.76	−6.41	−3.87	−4.17
EPB	10.75	8.65	9.32	9.67
ENPOLAR	−1.2	−2.79	−3.47	−2.43
Total Binding Energy	−30.05	−39.62	−34.89	−37.18

## Conclusion

This study investigated the anti-cancer potential of NSP-B using a comprehensive strategy that combined network pharmacology, quantum polarized ligand docking, molecular simulation, and binding free energy calculation. The results of our study revealed that FGF6, FGF20, FGF22, and FGF23 are crucial biomarker proteins that NSP-B specifically targets for the therapy of cancer. By utilizing a quantum-polarized docking method, we were able to detect strong interactions between NSP-B and the critical hotspot residues of these target proteins. In addition, molecular simulations unveiled the stable dynamic behavior, favorable structural packing, hydrogen bonding, and flexibility of residues within each complex.

The computed binding free energy findings highlight the remarkable pharmacological characteristics of NSP-B in relation to FGF6, FGF20, FGF22, and FGF23. These collective insights strongly endorse the potential of NSP-B for further advancement as an anti-cancer medication, highlighting its promising suitability in furthering cancer treatment efforts. The study lacks experimental validation, and the predicted interactions and binding affinities need to be confirmed through laboratory experiments. To enhance the credibility of the findings, future research should aim to integrate computational results with experimental validation to provide a more comprehensive understanding of NSP-B’s anti-cancer potential.

## Data Availability

The raw data supporting the conclusion of this article will be made available by the authors, without undue reservation.
